# Supplementary material for: Sofigait—A Wireless Inertial Sensor-Based Gait Sonification System
Source: Sensors (Basel). 2022 Nov 14;22(22):8782. doi: 10.3390/s22228782 (PMC9698922; doi:10.3390/s22228782)
Supplement: Supplementary file 1 [file sensors-22-08782-s001.zip › Code S1.pdf]

Load packages

```
import numpy as np
import pandas as pd
import scipy as sp
import xlrd
import matplotlib.pyplot as plt
from scipy.signal import savgol_filter
from scipy.signal import argrelextrema
```

ID

```
id = input('Bitte eine ID für den Datensatz vergeben')
```

Dataload

```
# path1 sind sofidaten und path2 sind vicondaten

def s_dataimport(path):
  data = xlrd.open_workbook(path)
  sheet = data.sheet_by_index(0)
  winkel_l = np.asarray([sheet.cell(i, 13).value for i in range(1, shee
t.nrows)])
  winkel_r = np.asarray([sheet.cell(i, 14).value for i in range(1, shee
t.nrows)])
  return winkel_l, winkel_r

def v_dataimport(path):
  data = xlrd.open_workbook(path,encoding_override = "utf-8")
  sheet = data.sheet_by_index(0)
  winkel_l = np.asarray([sheet.cell(i, 2).value for i in range(5, 2500)
])
  winkel_r = np.asarray([sheet.cell(i, 5).value for i in range(5, 2500)
])
  return winkel_l, winkel_r

path1 = input('Bitte den Dateipfad für Sofigait angeben')
path2 = input('Bitte den Dateipfad für Sofigait angeben')

s_winkel_l, s_winkel_r = s_dataimport(path1)
v_winkel_l, v_winkel_r = v_dataimport(path2)

for element in v_winkel_l:
  element = float(element)

for element in v_winkel_r:
  element = float(element)
```

Filter sofigait data

```
cutoff = 6
sampling = 100
a,b = sp.signal.butter(2, 0.12, analog = False)

s_winkel_l_fb = sp.signal.filtfilt(a,b, s_winkel_l)
s_winkel_r_fb  = sp.signal.filtfilt(a,b, s_winkel_r)
```

Define and extract region of intrest

```
# define region of interest and cutting the curve by detection of all M
ax 2 within the filtered curve and then define cutted curve by the max2
 indices

setstart = int(input('Bitte den Startpunkt der Analyse eingeben (Immer
der letzte große Peak nach dem dann der Tiefpunkt das erste Min1 ist?')
)
cycles = int(input('wie viele Gangzyklen sollen ausgewertet werden?'))

def cut_curve(links, rechts, a, b):
  peaks_l = sp.signal.find_peaks(links, height=50)
  winkel_l_cut = links[peaks_l[0][a-1]:peaks_l[0][a+b]]
  peaks_r = sp.signal.find_peaks(rechts, height=50)
  winkel_r_cut = rechts[peaks_r[0][a-1]:peaks_r[0][a+b]]
  winkel_l_cut, winkel_r_cut

  return peaks_l, peaks_r, winkel_l_cut, winkel_r_cut

s_peaks_l, s_peaks_r, s_cut_l, s_cut_r = cut_curve(s_winkel_l_f, s_wink
el_r_f, setstart, cycles)
v_peaks_l, v_peaks_r, v_cut_l, v_cut_r = cut_curve(v_winkel_l, v_winkel
_r, setstart, cycles)
```

Find and list local extrema

```
# Die anderen Extrema finden. Nicht schön als Endlösung da das nur unte
r der Voraussetzung klappt, dass es eine gerade Anzahl an Peaks ist und
 alle gefunden wurden

def findpeaks(links, rechts):
  maxima_l = list(links[argrelextrema(links, np.greater, order=10)][:-
1])
  maxima_r = list(rechts[argrelextrema(rechts, np.greater, order=10)][:
-1])
  minima_l = list(links[argrelextrema(links, np.less, order=10)][:-2])
  minima_r = list(rechts[argrelextrema(rechts, np.less, order=10)][:-
2])

  max_1_l = maxima_l[::2]
  max_2_l = maxima_l[1::2]
  min_1_l = minima_l[::2]
  min_2_l = minima_l[1::2]
  max_1_r = maxima_r[::2]
  max_2_r = maxima_r[1::2]
  min_1_r = minima_r[::2]
  min_2_r = minima_r[1::2]

  return max_1_l, max_2_l, min_1_l, min_2_r, max_1_r, max_2_r, min_1_r,
 min_2_r


s_max_1_l, s_max_2_l, s_min_1_l, s_min_2_l, s_max_1_r, s_max_2_r, s_min
_1_r, s_min_2_r  = findpeaks(s_cut_l, s_cut_r)
v_max_1_l, v_max_2_l, v_min_1_l, v_min_2_l, v_max_1_r, v_max_2_r, v_min
_1_r, v_min_2_r  = findpeaks(v_cut_l, v_cut_r)
```

Mean Values of Events

```
s_mean_max_1_l = float(np.mean(s_max_1_l))
s_mean_max_2_l = float(np.mean(s_max_2_l))
s_mean_min_1_l = float(np.mean(s_min_1_l))
s_mean_min_2_l = float(np.mean(s_min_2_l))
s_mean_max_1_r = float(np.mean(s_max_1_r))
s_mean_max_2_r = float(np.mean(s_max_2_r))
s_mean_min_1_r = float(np.mean(s_min_1_r))
s_mean_min_2_r = float(np.mean(s_min_2_r))

v_mean_max_1_l = float(np.mean(v_max_1_l))
v_mean_max_2_l = float(np.mean(v_max_2_l))
v_mean_min_1_l = float(np.mean(v_min_1_l))
v_mean_min_2_l = float(np.mean(v_min_2_l))
v_mean_max_1_r = float(np.mean(v_max_1_r))
v_mean_max_2_r = float(np.mean(v_max_2_r))
v_mean_min_1_r = float(np.mean(v_min_1_r))
v_mean_min_2_r = float(np.mean(v_min_2_r))


s_means = [round(s_mean_max_1_l, ndigits = 2), round(s_mean_max_2_l,ndi
gits = 2), round(s_mean_min_1_l,ndigits = 2), round(s_mean_min_2_l,ndig
its = 2),
          round(s_mean_max_1_r,ndigits = 2), round(s_mean_max_2_r,ndigi
ts = 2), round(s_mean_min_1_r,ndigits = 2), round(s_mean_min_2_r, ndigi
ts = 2)]
v_means = [round(v_mean_max_1_l, ndigits = 2), round(v_mean_max_2_l,ndi
gits = 2), round(v_mean_min_1_l,ndigits = 2), round(v_mean_min_2_l,ndig
its = 2),
          round(v_mean_max_1_r,ndigits = 2), round(v_mean_max_2_r,ndigi
ts = 2), round(v_mean_min_1_r,ndigits = 2), round(v_mean_min_2_r, ndigi
ts = 2)]

Labels = ['Min1_l', 'Max1_l', 'Min2_l', 'Max2_l','Min1_r', 'Max1_r', 'M
in2_r', 'Max2_r']


List_of_means = pd.DataFrame(list(zip(s_means,v_means)), columns= ['sof
igait', 'Vicon'], index = Labels)

pd.DataFrame(list(zip(s_means,v_means)), columns= ['sofigait', 'Vicon']
, index = Labels)
```

Cut curves into gait cycles

```
def snip_curve(links, rechts):

# liste der Indices der Min_1 erstellen (sind die Schnittstellen) mit e
inem Mehr weil endpunkt definiert sein muss
  cutting_point_l = list(argrelextrema(links, np.less, order=20)[0][::2
])
  cutting_point_r = list(argrelextrema(rechts, np.less, order=20)[0][::
2])

  start = 0
  zyklen_l = []
  zyklen_r = []
# für die anzahl an cutting points werden Unterarrays erstellt die jewe
ils einen Gangzyklus enthalten und in einer Liste gebündelt

  for i in range(start, (len(cutting_point_l)-1)):
    zyklen_l.append(links[cutting_point_l[start]:(cutting_point_l[start
+1])-1])
    zyklen_r.append(rechts[cutting_point_r[start]:(cutting_point_r[star
t+1])-1])
    start = start + 1

  return zyklen_l, zyklen_r

s_snip_l, s_snip_r = snip_curve(s_cut_l, s_cut_r)
v_snip_l, v_snip_r = snip_curve(v_cut_l, v_cut_r)
```

extrapolate to 100 datapoints

```
def normalize_to_100(links, rechts):

  l_norm=[]
  for i in range (0,(len(links))):
    y = links[i]
    x = np.asarray(np.arange(0,len(links[i])))
    xnorm = np.linspace(min(x),max(x),100)
    left_norm = np.interp(xnorm, x,y)
    l_norm.append(left_norm)

  r_norm=[]
  for i in range (0,(len(rechts))):
    y = rechts[i]
    x = np.asarray(np.arange(0,len(rechts[i])))
    xnorm = np.linspace(min(x),max(x),100)
    right_norm = np.interp(xnorm, x,y)
    r_norm.append(right_norm)

  return l_norm, r_norm
```

```
s_norm_l, s_norm_r = normalize_to_100(s_snip_l, s_snip_r)
v_norm_l, v_norm_r = normalize_to_100(v_snip_l, v_snip_r)
```

Compute mean curves

```
def mean_curve (links, rechts):

  for i in range (0,(len(links))):
    mean_curve_l = np.mean(links, axis = 0)

  for i in range (0,(len(rechts))):
    mean_curve_r = np.mean(rechts, axis = 0)

  return mean_curve_l, mean_curve_r

s_mean_curve_l, s_mean_curve_r = mean_curve(s_norm_l, s_norm_r)
v_mean_curve_l, v_mean_curve_r = mean_curve(v_norm_l, v_norm_r)
```

Export data into cvs files

```
from pandas.core.frame import DataFrame
labels = ['sofi_links', 'sofi rechts', 'Vicon links', 'Vicon rechts']
exportdata = {labels[0]:s_mean_curve_l, labels[1]:s_mean_curve_r, label
s[3]:v_mean_curve_l, labels[3]:v_mean_curve_r}

Winkel = pd.DataFrame(data = exportdata)
csv1 = Winkel.to_csv(str(id)+'.csv',index=False)
csv2 = List_of_means.to_csv(str(id)+'_means.csv')
```

Datareshape from cvs

```
from main import ids,sofi_links, sofi_rechts, vicon_links,vicon_rechts

labels = ids

def Datenexport(datensatz):
    new_labels = []
    for element in labels:
        name, extension = os.path.splitext(element)
        new_labels.append(name)

    exportdata = {}
    i = 0
    for element in new_labels:
        key = element
        value = datensatz[i]
        i = i+1
        eintrag = {key:value}
        exportdata.update(eintrag)

    Winkel = pd.DataFrame(data = exportdata).T

    return Winkel

Daten_1 = Datenexport(sofi_links)
Daten_2 = Datenexport(sofi_rechts)
Daten_3 = Datenexport(vicon_links)
Daten_4 = Datenexport(vicon_rechts)

csv1 = Daten_1.to_csv('sofi_links.csv',index=True)
csv2 = Daten_2.to_csv('sofi_rechts.csv',index=True)
csv3 = Daten_3.to_csv('vicon_links.csv',index=True)
csv4 = Daten_4.to_csv('vicon_rechts.csv',index=True)
```

Main SPM

```
from SPM import load_data

sofi_links, sofi_rechts, vicon_links, vicon_rechts, ids =
load_data('Kurven')


sofi_links = np.array(sofi_links).reshape(22,100)
sofi_rechts = np.array(sofi_rechts).reshape(22,100)
vicon_links = np.array(vicon_links).reshape(22,100)
vicon_rechts = np.array(vicon_rechts).reshape(22,100)


t_links = spm1d.stats.ttest_paired(sofi_links, vicon_links)
ti_links = t_links.inference(alpha=0.05, two_tailed=True)

t_rechts = spm1d.stats.ttest_paired(sofi_rechts, vicon_rechts)
ti_rechts = t_rechts.inference(alpha=0.05, two_tailed=True)


t_sofi = spm1d.stats.ttest2(sofi_links, sofi_rechts)
ti_sofi = t_sofi.inference(alpha = 0.05, two_tailed = True, interp=True)

t_vicon = spm1d.stats.ttest2(vicon_links, vicon_rechts)
ti_vicon = t_sofi.inference(alpha = 0.05, two_tailed = True, interp=True)
```
