# Supplementary material for: Sofigait—A Wireless Inertial Sensor-Based Gait Sonification System
Source: Sensors (Basel). 2022 Nov 14;22(22):8782. doi: 10.3390/s22228782 (PMC9698922; doi:10.3390/s22228782)
Supplement: Supplementary file 1 [file sensors-22-08782-s001.zip › Participant_Rating S1.pdf]

Sonification Rating (English translation)

Did the sound of the feedback match the walking?

not at all ☐ ☐ ☐ ☐ ☐ ☐ very good

How well was it possible to separate the feedback for the left and right leg?

not at all ☐ ☐ ☐ ☐ ☐ ☐ very good

Could gait asymmetry be perceived by the feedback?

not at all ☐ ☐ ☐ ☐ ☐ ☐ very good

How pleasant or unpleasant was the acoustic feedback generally perceived?

unpleasant ☐ ☐ ☐ ☐ ☐ ☐ pleasant
