# Supplementary figures and images for: Sofigait—A Wireless Inertial Sensor-Based Gait Sonification System
Source: Sensors (Basel). 2022 Nov 14;22(22):8782. doi: 10.3390/s22228782 (PMC9698922; doi:10.3390/s22228782)

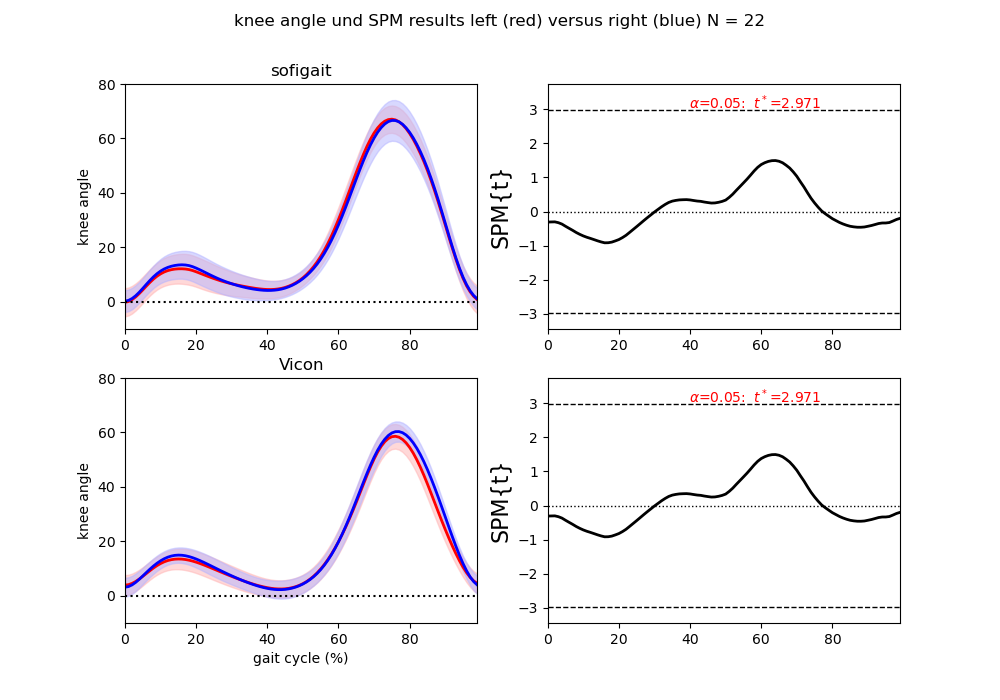

Supplement: Supplementary file 1 [file sensors-22-08782-s001.zip › Figure_S1.jpeg]
